# Door-to-antibiotic Time and In-hospital Mortality of Elder Patients Presenting to Emergency Department with Sepsis; a Cross-Sectional Study

**DOI:** 10.22037/aaem.v9i1.1266

**Published:** 2021-06-11

**Authors:** Siriwimon Tantarattanapong, Thanaporn Hemwej

**Affiliations:** 1 Department of Emergency Medicine, Songklanagarind Hospital, Faculty of Medicine, Prince of Songkla University, Hat Yai, Songkhla 90110, Thailand.

**Keywords:** Anti-Bacterial Agents, Mortality, Emergency Service, Hospital, Quality of Health Care, Sepsis; Aged, Aged, 80 and over

## Abstract

**Introduction:**

The current international sepsis guideline recommends that administration of intravenous broad-spectrum antibiotics should be initiated within 1 hour of emergency department (ED) arrival for sepsis patients. This study aimed to evaluate the association between door-to-antibiotic time and in-hospital mortality of these patients.

**Methods:**

In this retrospective cross-sectional study, elderly patients (age ≥65 years) diagnosed with sepsis in the ED of a tertiary referral and academic hospital from January to December 2019 were enrolled. Door-to-antibiotic time was defined as the time from ED arrival to antibiotic initiation. The associations of door-to-antibiotic time and each hour delay in first antibiotic initiation with in-hospital mortality were assessed.

**Results:**

Six hundred patients with the median age of 78.0 (IQR: 72.0-86.0) were studied (50.8% female). The median door-to-antibiotic time was 51.0 (36.0 – 89.0) minutes and in-hospital mortality rate was 12.5%. There was no significant difference in the in-hospital mortality rate between door-to-antibiotic time ≤1 hour and >1 hour (13.1% vs. 11.6%, p = 0.726). When considering hour-upon-hour of door-to-antibiotic time, no significant difference in in-hospital mortality was observed (p = 0.866). Factors that led to a delay in door-to-antibiotic time were presenting body temperature <38°C (odds ratio [OR] 3.34; 95% CI, 2.12-5.29; p < 0.001) and age <75 years (OR 1.7; 95% CI, 1.09-2.64; p = 0.019).

**Conclusion:**

Door-to-antibiotic time was not associated with in-hospital mortality in elderly sepsis patients in this study. Significant factors that led to a delay in door-to-antibiotic time were no fever, age <75 years, doctor time, and blood sample taking time.

## 1. Introduction

Sepsis remains as a public health problem worldwide and is one of the leading causes of death (1). The incidence of sepsis was 437 cases per 100,000 person-years and has tended to increase as a result of an aging population and more comorbidities (2). Sepsis is also the major cause of emergency department (ED) visits. The annual incidence of ED sepsis was 0.82% of total ED visits (3). Elderly patients constitute 60% of all sepsis patients (4). Likewise, elderly patients are an increasing proportion of ED visits in the range of 12-24% (5).

The characteristics of the elderly, such as unclear complaints, frequent hospital admission, increased cost and more resources used for care, and a higher rate of mortality, are different compared with younger patients (5). Particularly in institutionalized patients, up to a quarter of the elderly visit the ED with geriatric syndromes (5, 6). Therefore, reaching a diagnosis is challenging for the clinicians because nonspecific clinical manifestations of infection are common in such patients (7). Common presentations in the elderly include altered mental status, failure to eat and drink, failure to develop fever, lack of pain, functional decline, reduced mobility, falling, fatigue, and urinary incontinence (8). The absence of fever with a disease that is known to cause fever was the most common atypical presentation (9) and was associated with lower rates of ED antibiotic administration and mean intravenous (IV) fluid volume, and a higher rate of in-hospital mortality (10).

Many studies reported worse outcomes in delayed antibiotic administration in patients with sepsis and septic shock (11-15). The Surviving Sepsis Campaign (SSC) 2018 (16) strongly recommends initiating administration of IV broad-spectrum antibiotics within 1 hour of ED arrival as well as obtaining blood cultures, assessing serum lactate level, and initiating IV fluid resuscitation and early vasopressor treatment.

However, according to the Infectious Diseases Society of America (IDSA) (17), prescribing aggressive antibiotics and administrating antibiotics over a fixed time period may lead to deleterious consequences. Also, some studies and a meta-analysis reported no significant hour-upon-hour increase in sepsis mortality (18-20). Moreover, effective use of SSC protocols to administer antibiotics within 1 hour from ED arrival is challenging, especially in elderly patients. 

Therefore, what is the appropriate or suitable time for administration of antibiotics in elderly septic patients in the ED? The aim of this study was to determine whether the time of antibiotic initiation was associated with in-hospital mortality. We also aimed to determine the factors that led to delay in antibiotic initiation.

## 2. Methods

***2.1. Study design and setting***

This single-center retrospective cross-sectional study was conducted in elderly septic patients who visited the ED at Songklanagarind Hospital, which is a tertiary referral and academic hospital on the campus of Prince of Songkla University in southern Thailand. Approximately 50,000 patients visit the ED of this hospital each year. Patient data from January to December 2019 were retrieved from the hospital electronic medical record database. The Research Ethics Committee of the Faculty of Medicine, Prince of Songkla University approved the study (REC 62-425-20-4). 

***2.2. Participants***

The study enrolled elderly patients (≥65 years) diagnosed with sepsis at the ED according to the Songklanagarind Hospital sepsis protocol, which was developed from the recommendation of the SSC (16). Sepsis is defined as having both a suspected infection and an assessment of physiologic parameters using the National Early Warning Score (NEWS) of ≥5 points. In this study, infection was defined as a demonstrated source of infection or positive culture. The exclusion criteria were no sepsis in final diagnosis, referred patients, and incomplete data (e.g., serum lactate level and culture results). 

***2.3. Data gathering***

The data obtained from the electronic medical records included clinical presentation, demographic characteristics, Emergency Severity Index (ESI) triage level, initial NEWS, laboratory results (complete blood count [CBC], lactate values), treatments (antibiotics, IV fluid, oxygen therapy, mechanical ventilator, vasopressor), sources of infection, ED disposition, length of stay, and hospital outcome.

The door-to-antibiotic time was defined as the time from ED arrival to antibiotic initiation. Doctor time was the time elapsed until the doctor examined the patient. Hemoculture time and CBC time were the times when the nurse took blood samples. The time to receive IV fluids, time to receive vasopressor, time to start mechanical ventilation, and ED disposition time were recorded by the ED nurse. Fever was defined as body temperature ≥38°C.

***2.4. Outcome measurement***

The primary outcome was the association between the door-to-antibiotic time and in- hospital mortality among elderly patients. The in-hospital mortality rate was calculated in hospitalized sepsis patients. This study also analyzed and compared mortality and door-to-antibiotic time interval as ≤1 hour and each hour interval beyond the first hour. The secondary outcomes were the factors that affected the delay in antibiotic initiation and the most prevalent sources of infection in elderly sepsis patients who visited the ED.

***2.5. Statistical analysis***

The n4Studies was used to calculate the sample size using a two-tailed test based on a study by Tongnoon (21). The final sample size was 534 patients to allow for an expected drop-out rate of 10%. Continuous data are demonstrated as median with interquartile range (IQR) or mean ± standard deviation. Categorical data are presented as number and percentages. The Pearson’s chi-squared test was performed on categorical data for the primary outcome. The chi-square test was used for the analysis and to compare mortality and door-to-antibiotic time interval at ≤1 hour and at each hour interval beyond the first hour. After testing associations, selected variables with p-values <0.2 were introduced into a multiple logistic regression model for secondary outcomes. Odds ratios (ORs) for the outcomes and their 95% confidence intervals (CIs) were used to identify the significant factors that led to delay in antibiotic initiation. A two-sided p-value <0.05 was considered statistically significant. All statistical analyses were performed using R software version 3.5.1 (R Foundation for Statistical Computing, Vienna, Austria). 

## 3. Results

***3.1. Characteristics of the study population***

**Figure 1:**
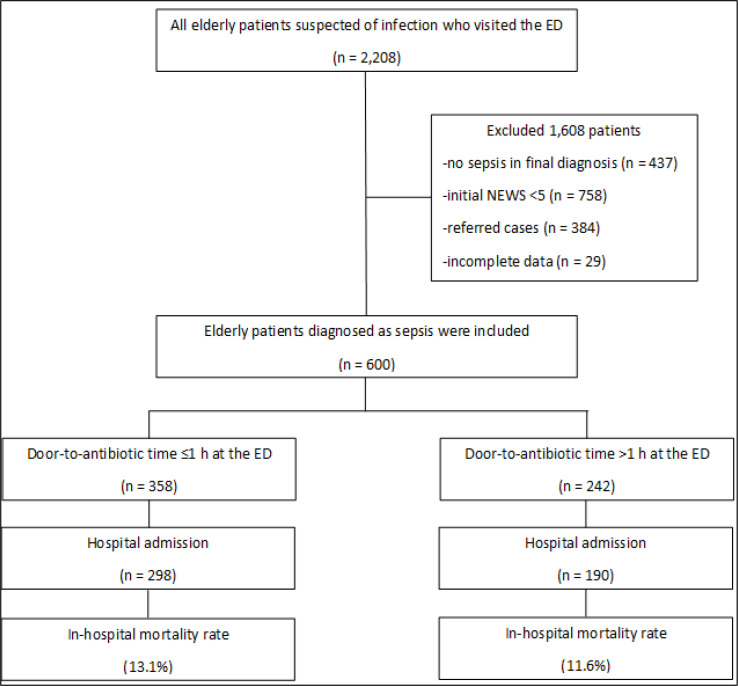
Flowchart of patients' enrollment

The files of 2,208 elderly patients suspected of infection were reviewed. The patients with no sepsis in the final diagnosis, initial NEWS <5 points, referred cases, and charts/files with incomplete data were excluded. The flowchart of the enrollment process is shown in Figure 1. A total of 600 patients with the median age of 78.0 (IQR: 72.0-86.0) years were included in the study (50.8% female). The most common sources of infection were pneumonia (38.2%), followed by urinary tract (23.2%), intra-abdominal (16.2%), bacteremia (14.7%), and skin/soft tissue (5.5%). Among intra-abdominal infections, acute gastroenteritis (8.3%), acute cholangitis (4.8%), and acute cholecystitis (1.2%) were the most common. Ninety-one patients (15.2%) had positive hemocultures for *Escherichia coli* (52.7%), *Klebsiella pneumoniae* (18.7%), *Staphylococcus spp.* (12.1%), and *Streptococcus spp.* (12.1%). Frequently prescribed empirical antibiotics were ceftriaxone (64.5%), piperacillin/tazobactam (20.8%), ceftazidime (8%), and carbapenems (4.8%).

The percentages of door-to-antibiotic times of ≤1 hour and >1 hour were 59.7% (358/600) and 40.3% (242/600), respectively. Table 1 and 2 compared the baseline characters, laboratory findings and outcomes between cases with door to antibiotic time of ≤ 1 and > 1 hour. A comparison between the two groups showed that the ≤1-hour group had a significantly higher ESI level and initial NEWS. The median NEWS was 8 points in the ≤1-hour group and 6 points in the >1-hour group. Patients in the ≤1-hour group also had more cerebrovascular diseases as co-morbidities and received more vasopressor agents and IV fluids in the ED. Patients who complained of fever and higher body temperature (BT), and had a change in the Glasgow Coma Scale score from baseline received antibiotic administration significantly early. Patients who complained of gastrointestinal symptoms received antibiotic administration significantly later. 

***3.2. Primary outcomes***

**Figure 2:**
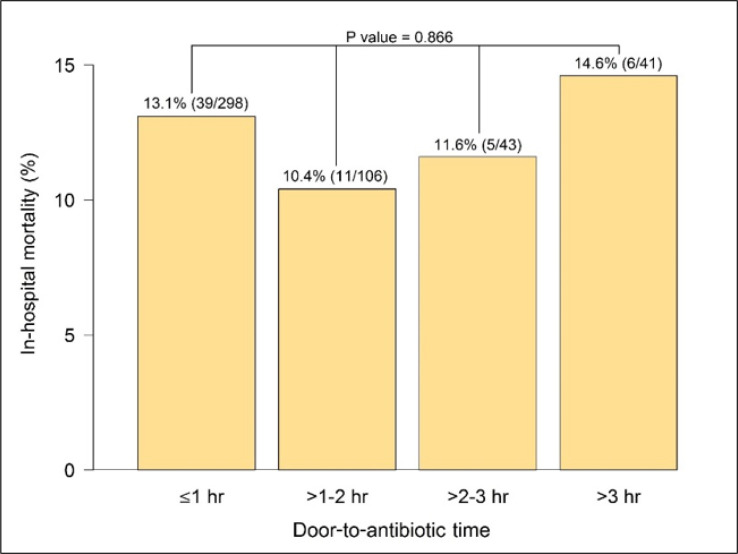
Percentages of in-hospital mortality and door-to-antibiotic time

The median (IQR) door-to-antibiotic time was 51.0 (36.0–89.0) minutes, and the in-hospital mortality rate was 12.5%. There was no significant difference in the median (IQR) door-to-antibiotic times between the discharged patients and those who died in the hospital, 51.0 (36.0–89.0) vs. 54.0 (41.0-85.0); p = 0.382). Similarly, there was no significant difference in the in-hospital mortality rate of those with door-to-antibiotic time of ≤1 hour and >1 hour (13.1% vs. 11.6%; p = 0.726). When considering hour-upon-hour of door-to-antibiotic time, no significant difference in the in-hospital mortality rate was observed (p = 0.866). However, the in-hospital mortality rates tended to show a linear increase when each extra hour of door-to-antibiotic time was considered independently. The in-hospital mortality rates of door-to-antibiotic times of 1-2 hours, 2-3 hours, and >3 hours were 10.4%, 11.6%, and 14.6%, respectively (Figure 2; p = 0.866).

***3.3. Secondary outcomes***

In multivariate analysis, delays in antibiotic initiation of >1 hour were associated with presenting body temperature <38.0°C (OR 3.34; 95% CI: 2.12-5.29; p < 0.001) and age <75 years (OR 1.70; 95% CI: 1.09-2.64; p = 0.019) (Table 3). 

## 4. Discussion

In this retrospective observational study of elderly patients with sepsis in the ED, door-to-antibiotic time was not associated with in-hospital mortality. Sterling et al. (18) found no significant differences when comparing the antibiotic administration within 3 hours from ED triage and within 1 hour from septic shock recognition. 

Door-to-antibiotic time and in-hospital mortality were the main focuses of this study, which showed that each extra hour (relative to door-to-antibiotic time ≤1 hour) was not associated with an increase in the mortality rate. The highest mortality rate in this study was in the door-to-antibiotic group of >3 hours. Likewise, Peltan et al. (22) found that a door-to-antibiotic time cutoff of 3 hours was associated with mortality, but a cutoff of 1 hour did not show statistical significance. When the door-to-antibiotic times of ≤1 hour and >1 hour were compared, the ≤1-hour group had greater severity of illnesses based on the ESI level and NEWS. For this reason, the door-to-antibiotic time of ≤1 hour had a higher mortality rate than the patients who received antibiotics later.

The SSC guideline recommends antibiotic initiation within 1 hour. Nonetheless, many studies showed failure to achieve that goal. For instance, Abe et al. (23) found that 30.5% of cases received antibiotics within 1 hour. Ko et al. (24) revealed that the 1-hour target was achieved in 28.6% of septic shock patients treated in the ED. In this study, 59.7% of the patients received antibiotics within 1 hour. The explanation of the differences is that the protocol used to diagnose sepsis was different from the other reports. The median door-to-antibiotic time in this study was 54 minutes, which was shorter than a former report (119 minutes) (25). The reason was a different set of criteria for a diagnosis of sepsis and our standard care followed the hospital policy. 

Overall, the in-hospital mortality rate was 12.5%, which differed from the other studies in elderly patients with sepsis. Martin-Loeches et al. (26) found that the overall hospital mortality was 48.8% and Vardi et al. (27) found a 38.4% mortality rate in patients older than 85 years. The mortality rate in this study was much lower. The explication is that this study collected data in the ED, while the previous studies collected data in the intensive care unit (ICU) where the patients had more severe conditions.

Atypical presentation accounts for about a third of elderly patients in the ED and a lack of fever is common in the elderly (9). It was found that 29.7% of elderly patients with sepsis have no fever on arrival. Henning et al. (10) found that afebrile patients with septic shock in the ED had an increased likelihood of in-hospital mortality compared with febrile patients. Similarly, Rumbus (28) reported that septic patients with normothermia had a higher mortality rate (31%) compared to those with fever (22%). In this study, no fever (BT <38°C) was an independent factor that led to delay in antibiotic initiation.

Pneumonia, urinary tract infections, and intra-abdominal infections were found to be the three most common sources of infection in elderly patients. This finding was similar to studies previously reported in Thailand (29, 30).

Emergency physicians should be careful when evaluating the elderly with sepsis by keeping in mind that most elderly patients have atypical presentations. Age <75 years, body temperature <38°C, doctor time, and blood sample taking time were significant factors that led to delay in antibiotic initiation.

This study showed that door-to-antibiotic time was not associated with in-hospital mortality in elderly sepsis patients. This result supported the IDSA recommendation. For sepsis in elderly patients who present with non-specific symptoms or geriatric syndromes, taking time to perform appropriate investigations may be reasonable, because appropriate and smart antibiotic use is an important issue.

**Table 1:** Comparing the baseline characteristics between cases with door-to-antibiotic time of ≤ 1 and > 1 hour

**Characteristics**	**Door-to-antibiotic time (hour)**		**p-value**
	**≤1 (n=358)**	**>1 (n=242)**	
**Age (year)**	80.0 (73.0- 86.0)	77.0 (70.0-84.0)	0.014
**Gender**			
Female	176 (49.2)	129 (53.3)	0.361
Male	182 (50.8)	113 (46.7)	
**ESI levels**			
1	67 (18.7))	32 (13.2)	<0.001
2	264 (73.7)	143 (59.1)	
3	25 (7.0)	66 (27.3)	
4	2 (0.6)	1 (0.4)	
**Co-morbidities**			
Diabetes mellitus	127 (35.5)	73 (30.2)	0.206
Hypertension	180 (50.3)	122 (50.4)	1.000
Chronic kidney disease	54 (15.1)	37 (15.3)	1.000
Cerebrovascular disease	115 (32.1)	51 (21.1)	0.004
Heart disease	77 (21.5)	54 (22.3)	0.894
Respiratory disease	57 (15.9)	51 (21.1)	0.133
Malignancy	85 (23.7)	52 (21.5)	0.585
**Medication use**			
Systemic steroid	30 (8.4)	14 (5.8)	0.300
Immunosuppressive agents	4 (1.1)	5 (2.1)	0.496
Chemotherapy (within 1 month)	23 (6.4)	15 (6.2)	1.000
Beta blocker	76 (21.2)	41 (16.9)	0.232
Bronchodilator	43 (12)	32 (13.2)	0.753
Central acting agents	53 (14.8)	33 (13.6)	0.778
Psychotropic medication	25 (7)	15 (6.2)	0.833
Opioids	16 (4.5)	7 (2.9)	0.441
**Chief complaint**			
Fever	180 (50.3)	98 (40.5)	0.023
Respiratory tract symptoms	91 (25.4)	62 (25.6)	1.000
Drowsy, stupor, coma	35 (9.8)	26 (10.7)	0.805
Gastrointestinal symptoms	33 (9.2)	41 (16.9)	0.007
Fatigue	12 (3.4)	6 (2.5)	0.711
Fall	3 (0.8)	2 (0.8)	1.000
**Presenting vital signs**			
Body temperature (°C)	38.5 (38.0-39.1)	38.1 (37.0-38.8)	<0.001
Pulse rate (/ minute)	108.3 ± 20.7	106.0 ± 18.2	0.160
Systolic blood pressure (mmHg)	135.5 ± 31.8	135.4 ± 28.6	0.951
Respiratory rate (/minute)	32.0 (28.0-36.0)	30.0 (26.0-36.0)	0.019
GCS change from baseline	89 (24.9)	41 (16.9)	0.027
**Initial NEWS**	8 (6-10)	6 (5-8)	<0.001

Data are presented as n (%), mean ± standard deviation or median and interquartile range (IQR). ESI: Emergency Severity Index; NEWS: National Early Warning Score; GCS: Glasgow Coma Scale.

**Table 2:** Comparing the laboratory findings and outcomes between cases with door-to-antibiotic time of ≤ 1 and > 1 hour

**Characteristics**	**Door-to-antibiotic time (hour)**		**P**
	**≤1 h (n=358)**	**≤1 h (n=358)**	
**ED laboratory findings**			
WBC (1,000/dL)	11.6 (8.2-16.1)	11.4 (8.1-15.9)	0.973
PMN (%)	82.0 (73.3-88)	83.4 (74.8-89)	0.087
Band cells (%)	6.0 (2.0-13.0)	3.0 (1.0-11.0)	0.013
Lactate ≥2 mmol/L	145.0 (44.5)	73.0 (38.8)	0.248
**ED treatment**			
Vasopressor	34 (9.5)	11 (4.5)	0.036
IV fluid replacement (CC)	298 (83.2)	162 (66.9)	<0.001
**ED length of stay (minute)**	265.0 (211.5-350.8)	290.5 (218-369.8)	0.133
**ED disposition**			0.171
Intensive care unit	30 (8.4)	16 (6.6)	
Ward	213 (59.5)	125 (51.7)	
Short-stay observation unit	55 (15.4)	49 (20.2)	
Discharge	59 (16.5)	50 (20.7)	
Death in ED	1 (0.3)	2 (0.8)	
**Outcome of admission**			
Discharge	259 (86.9)	168 (88.4)	0.726
Death	39 (13.1)	22 (11.6)	
Hospitalization (days)	7.0 (3.0–14.0)	6.5 (3.0-11.8)	0.090
**Time**			
Door-to-doctor time (minute)	3.0 (0.0–7.0)	6.0 (2.0-14.0)	<0.001
Door-to-CBC time (minute)	29.0 (18.2-38.8)	44.0 (29.0–65.0)	<0.001
Door-to-lactate time (minute)	19.0 (10.0 -36.0)	36.0 (16.5-83.0)	<0.001

Data are presented as n (%), mean ± standard deviation or median and interquartile range (IQR). ED: Emergency department; WBC: white blood cell; PMN: polymorphic neutrophils; IV: intravenous; CBC: complete blood count.

**Table 3:** Multiple logistic regression analysis of factors that led to a delay in antibiotic initiation (>1 hour)

**Variables**	**Adjusted OR **	**95% CI**	**p-value**
No fever (BT <38°C)	3.34	2.12 - 5.29	<0.001
Age <75 years	1.70	1.09 - 2.64	0.019
Door-to-doctor time	1.04	1.01 - 1.06	0.002
Door-to-CBC time	1.04	1.03 - 1.05	<0.001
Door-to-lactate time	1.01	1.00 - 1.01	0.006

OR: odds ratio; CI: confidence interval; BT: body temperature; CBC: complete blood count.

## 5. Limitations

We acknowledge that this study has several limitations. First, this study was retrospective in nature, which resulted in selection bias; because patients with incomplete data were excluded. Second, we could not determine the direct cause and effect, which could subject the study to confounding, and there were uncertain factors that possibly made causal inference between antibiotic time and in-hospital mortality. Third, this study used the Songklanagarind Hospital sepsis protocol and criteria for the diagnosis of sepsis that are probably different from other institutions, which may limit generalizability. Fourth, the antibiotic times and hospital in-hospital mortality were not evaluated to arrive at an adjusted severity of sepsis. Thus, the findings of this study should be applied with caution in septic shock patients.

## 6. Conclusions

Door-to-antibiotic time was not associated with in-hospital mortality in elderly sepsis patients in this study. In addition, a linear association between each hour of delay in first antibiotic initiation and in-hospital mortality was not observed. 

## 7. Declarations

### 7.1. Author contributions

Thanaporn Hemwej performed the literature search, study design, data collection, data analysis, data interpretation, and wrote the manuscript. Siriwimon Tantarattanapong did the literature search, study design, critical revision and wrote the manuscript.

### 7.2. Acknowledgments

The authors are grateful to Ms. Kingkarn Waiyanak for article searches and retrieval, Ms. Nannapat Pruphetkaew, Epidemiology Unit, Faculty of Medicine, Prince of Songkla University for statistical assistance, and Glenn K. Shingledecker for his help in editing the manuscript.

### 7.3. Conflict of interest

The authors report no conflicts of interest in this work.

### 7.4. Funding and support

The Faculty of Medicine, Prince of Songkla University funded this research.
